# Emission Enhancement of Fluorescent Molecules by Antireflective Arrays

**DOI:** 10.34133/2019/3495841

**Published:** 2019-11-27

**Authors:** Hongbo Xu, Lingxiao Liu, Fei Teng, Nan Lu

**Affiliations:** ^1^MIIT Key Laboratory of Critical Materials Technology for New Energy Conversion and Storage, School of Chemistry and Chemical Engineering, Harbin Institute of Technology, 150001 Harbin, China; ^2^State Key Laboratory of Supramolecular Structure and Materials, College Chemistry, Jilin University, 130012 Changchun, China

## Abstract

Traditional fluorescence enhancement based on a match of the maximum excitation or emission of fluorescence molecule with the spectra of the nanostructure can hardly enhance blue and red fluorescent molecules. Here, an enhanced method which is a new strategy based on the antireflective array has been developed to enhance the emission of blue and red fluorescent molecules. The fluorescence emission is enhanced by increasing the absorption at excitation wavelengths of the fluorescent molecules and reducing the fluorescent energy dissipation with an antireflective array. By introducing the antireflective arrays, the emission enhancement of blue and red fluorescent molecules is, respectively, up to 14 and 18 fold. It is a universal and effective strategy for enhancing fluorescence emission, which could be applied to enhance the intensity of organic LED and imaging.

## 1. Introduction

Fluorescence sensing technique plays a more and more important role in optical imaging for environmental monitoring, disease diagnosis, genomic/proteomic research, etc., due to its advantages, such as simplicity, high sensitivity, and various organic dyes with diverse spectral properties [1–4]. However, the conventional approaches based on fluorescence are limited by the isotropy of fluorescence emission, which reduces the detection efficiency. Therefore, it is essential to enhance the fluorescence emission of fluorescent molecules for significantly improving their performance, such as sensitivity, quality of imaging, or a lower limit of detection (LOD) of the related systems [5].

To date, the most extensive methods to enhance fluorescence are to synthesize conjugated polymers or construct metal structures [6, 7]. The essence of these methods is to enhance the excitation, quantum efficiency (QE), and extraction [8]. The fluorescence is amplified by conjugated polymers through enhancing excitation and QE, but this method generally suffers from the high selectivity of the conjugated polymers, which only works with the specific fluorophores [9]. In addition, the synthesis procedure is complicated, which restricts this technique from practical applications. In order to achieve metal fluorescence enhancement, a unique plasma structure with a metal surface/nanoparticle can be constructed because this composite structure can change the optical properties of the local fluorophore [10]. However, the MEF method requires effective coupling with the emission of the fluorophores and the surface plasmon resonance of metallic nanostructures [11]. Therefore, it is still a challenge to enhance multispecies fluorescence molecules using one metal structure [12]. Meanwhile, the MEF method has a poor uniformity over a large area for the fluorescence detection due to the uneven dispersion of the “hot spots.”

Recently, fluorescence enhancement based on photonic crystals (PCs) through excitation enhancement and extraction enhancement has been demonstrated for fluorescence-based detection [13–18]. Near the stopband of PCs, light propagates at reduced group velocity owing to resonant Bragg scattering, which can enhance optical gain and lead to stimulated emission, as well as amplifying the excitation of the incident light [19–23]. In particular, the three-dimensional (3D) opal or inverse opal PCs can largely enhance the luminescence of dyes and be easily prepared. Many researchers have employed this method and achieved a series of successful results [24–27]. However, the enhancement requires that the maximum excitation or emission of fluorescence molecule should match with the photonic crystal reflection, which restricts these techniques from practical applications [28–30]. The antireflective arrays can enhance the absorption of light in a wide range of wavelengths due to reducing the surface reflective [31–36]. In addition, the antireflective arrays can enhance the solid angle of the escape cone of fluorescent film.

Based on the above considerations, this study reported a method for enhancing fluorescence emission using an antireflective array, which is applicable for different fluorescence molecules. The antireflective structures are fabricated by nanoimprint lithography (NIL) to create nanoholes on the film of PMMA with fluorescent molecules, which can dramatically suppress the surface reflection from visible to near-infrared regions. The fluorescence emission of different molecules can be enhanced on the same nanohole arrays.

## 2. Results and Discussion

**Figure 1:**
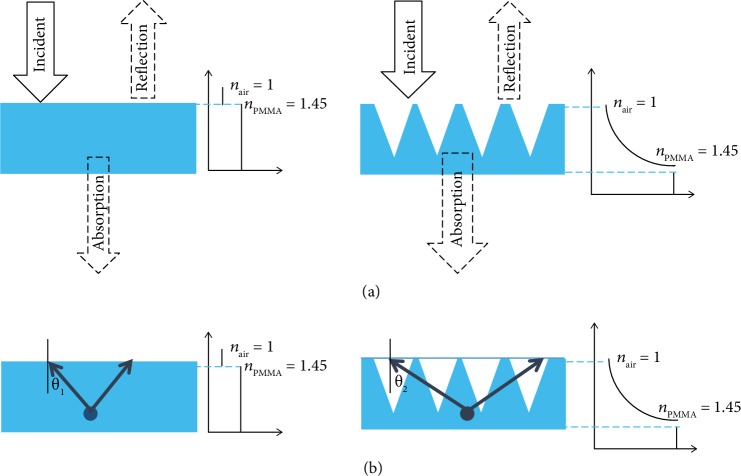
Schematics of mechanisms of fluorescent enhancement of a flat PMMA film and PMMA nanohole arrays, (a) schematics of antireflection, and (b) schematics of the critical angle.

The antireflective arrays can enhance fluorescent of dye due to enhancing the absorption and solid angle of the escape cone. In this work, we also employ this strategy to fabricate the antireflective array on the film of PMMA with fluorescent molecules.  Figure 1 shows schematics of mechanisms of fluorescent enhancement of a flat PMMA film and PMMA nanohole arrays. When the light wavelength is considered as an incident on a flat of PMMA surface, Fresnel reflection, in this case, occurs because of their differences in refractive index. The index (*n*) between PMMA (*n*_PMMA_) and air (*n*_air_) changes suddenly at their interface, which causes a relatively higher reflectivity of the flat PMMA intersurface. The nanohole on the PMMA replication surface to a certain degree could be approximate as a group of multiple effective dielectric layers, which possess gradient index from *n*_air_ to *n*_PMMA_. On the base of the medium theory, the refractive index (*n*_eff_) of the positive incidence through the nanohole on the PMMA replica to a certain degree could be written as equation (1). The *n*_eff_ changes gradually from 1.0 (*n*_air_) to 1.45 (*n*_PMMA_) along the nanohole. Therefore, the PMMA nanohole surface exhibits a lower reflectivity and higher absorption than the flat PMMA surface. According to the above results, the enhancement of fluorescence emission should be contributed by the increase of the absorption at excitation wavelengths of the fluorescent molecules because more energy can be used for the excitation of fluorescent molecules.

(1) $$\begin{array}{c} {\text{n}}_{\text{eff}}=\sqrt{\frac{\left(1-\text{f}+\text{f}{\text{n}}_{\text{PMMA}}^{2}\right)\left\{\text{f}+\left(1-\text{f}\right){\text{n}}_{\text{PMMA}}^{2}\right\}+{\text{n}}_{\text{PMMA}}^{2}}{2\left\{\text{f}+\left(1-\text{f}\right){\text{n}}_{\text{PMMA}}^{2}\right\}}}, \end{array}$$

where *n*_PMMA_ is regarded as the important refractive index of the structure and *f* is thought to a significant factor of the antireflective array, which is regarded as the main volume percentage of the antireflective array in this movie [34]. *f* is calculated from the information of the cross-sectional SEM; *f* is considered as a function of observing and calculating the height of the antireflection array.

The critical angle of total reflection of PMMA nanohole surface and flat PMMA surface is shown Figure 1(b). On the base of Snell's Law, the critical angle of total reflection (*θ*_1_) on the PMMA surface to a refractive index of air and PMMA film could be written as equation (2) [37]:

(2) $$\begin{array}{c} \theta_{1}=\sin^{-1}\frac{n_{\text{air}}}{n_{\text{substrate}}}, \end{array}$$

where *θ*_1_ is the critical angle of total reflection, *n*_substrute_ is regarded as the important refractive index of the structure, and *n*_air_ is the air refractive index. The *n*_substrate_ is calculated from the information of equation (1). The sharp contrast in reflectivity at 43.6° for this flat PMMA is observed in Figure 1(b). When light rays strike the flat-top surface at an angle larger than 43.6°, its reflectivity instantaneously rises to unity, corresponding to the critical angle at the interface between PMMA and air. On the base of equation (2), the critical angle can be determined as 43.6°. The critical angle of PMMA nanohole (*n*_eff_ = 1.05) can be determined as 73.7°, similarly. The critical angle of PMMA nanohole exhibits a higher angle than the flat PMMA surface (Figure 1(b)). According to the above results, contribution might come from the reduction of the fluorescent energy dissipation on the antireflective structure. The solid angle of the escape cone by the critical angle of luminescent thin films to a critical angle of air and PMMA nanostructure could be written as equation (3) [37]:

(3) $$\begin{array}{c} \Omega =\int_{0}^{\theta_{1}}\sin\theta d\theta =2\pi \left(1-\cos\theta_{1}\right), \end{array}$$

where *n*_substrate_ is the solid angle of the escape cone by the critical angle of luminescent thin films, *θ*_1_ is a critical angle of total reflection. According to equation (3), the ratio of the fluorescence intensity of PMMA nanohole and flat PMMA can be calculated. A measure of the final fluorescence enhancement equals to the product of increased absorption and increased solid angle of the escape cone.

**Figure 2:**
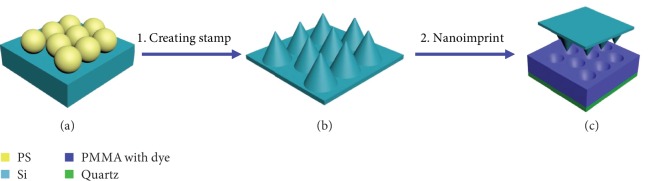
Fabrication process of the biomimetic antireflective nanohole array. (a) Preparation of the monolayer of PS nanospheres on Si substrates. (b) Creation of the Si stamp by RIE with nanospheres as a mask. (c) Fabrication of the biomimetic antireflective nanohole array with nanoimprint lithography.

Figure 2 schematically shows a process to fabricate nanohole antireflective arrays; we designed a simple nanostructure transfer process in a typical experimental. The Si stamps for fabricating antireflective arrays were prepared according to the reference [34]. Firstly, a monolayer of the hexagonal closed-packed PS spheres was prepared on Si (100) substrate (shown in [Supplementary-material supplementary-material-1]). Secondly, the Si stamp of antireflective arrays was fabricated by reactive ion etching (RIE). Finally, the antireflective array was fabricated by nanoimprinting the spin-coated film of PMMA mixed with dye molecules on a glass substrate. [Supplementary-material supplementary-material-1] shows the scanning electron microscopy (SEM) images of the fabricated Si nanopillar arrays. The periodicity of Si nanopillar arrays is consistent with that of the mask, which indicates that the mask patterns are faithfully transferred onto Si substrates using RIE. The height and shape of nanopillars can be controlled by varying the etching duration. As presented in [Supplementary-material supplementary-material-1], the height of the Si nanopillars is about 120 nm when the etching duration is 3 min. Upon extending the etching duration, the Si nanopillars increase in height and change from sphere to taper. As demonstrated in [Supplementary-material supplementary-material-1], the height of the Si nanopillars is 300 nm, 450 nm, 600 nm, and 780 nm when the etching duration is 5 min, 7 min, 9 min, and 11 min, respectively. Meanwhile, the top of Si nanopillars is changed from a sphere to a taper with extending the etching duration. The reason is that the diameter of PS spheres is reduced gradually by O_2_with extending the etching duration, which makes more surface of the top of Si nanopillars exposed to the etching gas and is etched. The hemispherical reflectivity comparison of the Si nanopillar arrays and the polished Si wafer in the waveband of 300-900 nm is shown in [Supplementary-material supplementary-material-1], which demonstrates that the Si nanopillar arrays efficiently suppress the reflection. The reflectivity of the Si slide in the range of 500 nm to 900 nm is suppressed from 30% to 5% when the height of the Si nanopillars increases from 120 nm to 780 nm. The reflectivity of the Si nanopillars decreases with the increase of the heights, as presented in [Supplementary-material supplementary-material-1]. According to the effective medium theory, [34], the Si nanopillar arrays can be considered as an effective medium layer, which provides a gradient refractive index and results in a reduction of reflectivity.

**Figure 3:**
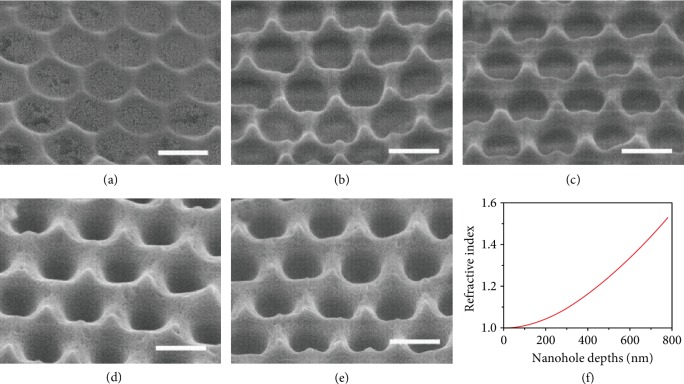
SEM images of the nanohole arrays with the depth of (a) 120 nm, (b) 295 nm, (c) 440 nm, (d) 585 nm, and (e) 750 nm. (f) Correlation of the refractive index and depths of nanohole arrays, the sample is shown in Figure 2(e). The scale bar represents 500 nm.

A fluorescent molecule with a blue emission, 2,5,20,50-tetrakis (2,2-diphenylvinyl)biphenyl (TDPVBi), whose excitation and emission wavelengths are 352 nm and 452 nm, was mixed with PMMA and spin-coated on a quartz slide to form a flat film. Then, the nanohole arrays with different depths were created by nanoimprint lithography. Figure 2 shows the SEM images of the fabricated nanohole arrays by using the stamps of different depths. The periodicity of nanohole arrays is consistent with the corresponding stamps, but the depths of the nanohole arrays are slightly lower than that of the corresponding Si stamps. The volume shrinkage of PMMA should introduce the difference after cooling down. As demonstrated in Figures 3(a)–3(d), the nanohole arrays with the depths of 120 nm, 295 nm, 440 nm, 585 nm, and 750 nm are created with the stamps of 120 nm, 300 nm, 450 nm, 600 nm, and 780 nm in height, respectively.  Figure 3(f) demonstrates the correlation of refractive index and array depths, the minimum refractive index of nanohole arrays is about 1.0, and the maximum refractive index is almost equal to the refractive index of the PMMA nanoholes with 750 nm in height.

**Figure 4:**
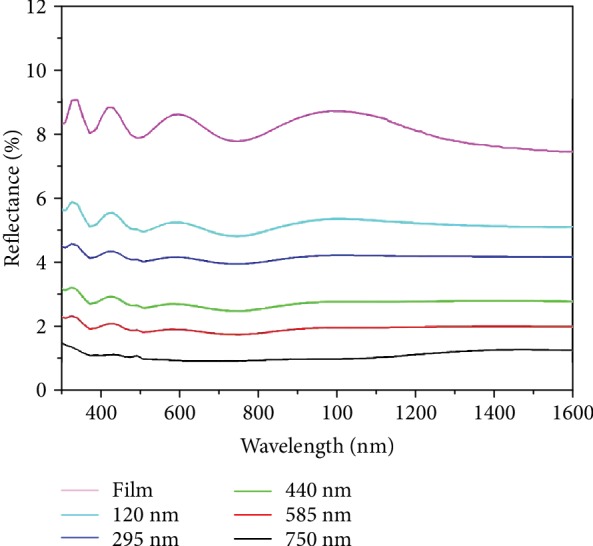
Hemispherical optical reflection of a flat film and the nanohole arrays with different depths at normal incidence.

Figure 4 shows the hemispherical reflectivity of a flat film and nanohole arrays in the range of 300-1600 nm at normal incidence. The average reflectivity of the nanohole arrays decreases from 5.5% to 1.5% when the depth of the nanohole arrays increases from 120 nm to 750 nm. From the effective medium theory, the tapered structure can decrease reflectivity to the minimum. However, the spacing between the PMMA nanoholes makes *n*_eff_ abruptly increase at the interfaces of substrate and air, which can introduce intense reflection. The correlation between *n*_eff_ and the depth of nanohole arrays is presented in Figure 3(f), which shows that the minimum *n*_eff_ of nanohole arrays is about 1.0, and the maximum *n*_eff_ is almost equal to the refractive index of the substrate, leading to a lower reflection.

**Figure 5:**
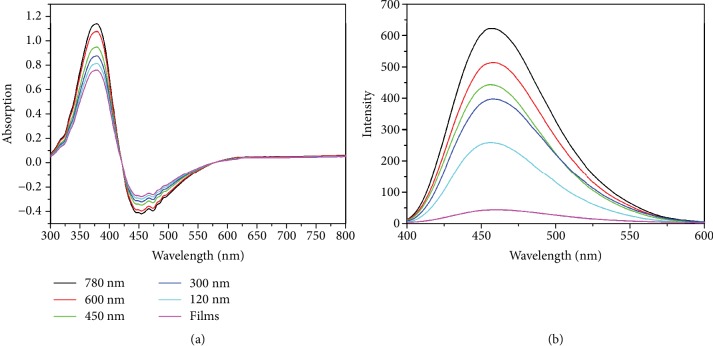
(a) Absorption and (b) fluorescence spectra collected on a flat film of TDPVBi mixed with PMMA, and the nanohole arrays generated on the mixed film.

To quantitatively characterize the absorption of the nanohole arrays, we carried out hemispherical measurements with an integrating sphere. A xenon lamp is coupled into the fiber and then spectroscopically measured using a Maya spectrometer. The sample was mounted at the center of the sphere. This xenon lamp was a continuous source with a wavelength range of 300 nm to 800 nm. The fluorescence of the sample was excited when the light entered the integrating sphere. The integrating sphere uniformly scattered the reflected, transmitted light and fluorescence from the sample and collected by a photo detector. In our measurement, the light is considered to be reflected from transmit through the sample and fluorescence so that the result can be considered as a measurement of the absolute absorption. Then, the spectra showed a negative absorption at the wavelength of the fluorescence due to the light energy increases. Meanwhile, this can be considered as a measurement of the fluorescence from samples. The total absorption measurement was conducted at the wavelengths of 300 nm to 800 nm, which covers most of the spectra for fluorescence sensing and imaging. The total absorption of TDPVBi at 352 nm is shown in Figure 5(a). The absorption of the nanohole arrays correspondingly increases from 0.68 to 1.1 when the reflectivity gradually drops from 9% to 1.2% at the wavelength of 352 nm. The absorption of TDPVBi on nanohole arrays is enhanced by reducing the reflectivity. The total absorption of TDPVBi at the wavelength of 452 nm is less than zero, as shown in Figure 5(a), which can be regarded as the emission of TDPVBi. As demonstrated in Figure 5(a), the fluorescence emission of the nanohole arrays is -0.24, -0.28, -0.31, -0.34, -0.38, and -0.42 at a wavelength of 452 nm when the absorption is 0.68, 0.79, 0.85, 0.9, 1.03, and 1.1 at a wavelength of 352 nm, respectively. The fluorescence emission of TDPVBi enhances with increasing absorption of the nanohole arrays. Figure 5(b) shows the fluorescent spectra collected on a flat film of TDPVBi mixed with PMMA and the nanohole arrays created on the above film. The fluorescence intensity of the flat film is about 48 at the wavelength of 452 nm, which increases from 200 nm to 680 nm when the depth of nanoholes increases from 120 nm to 750 nm. The emission enhancement of fluorescent with 750 nm nanohole is up to 14 times. According to equation (3), this fluorescence intensity with antireflective arrays is increased by 4 times. At the same times, combined with the increased absorption, the total fluorescence intensity with antireflective arrays is about 14 times.

**Figure 6:**
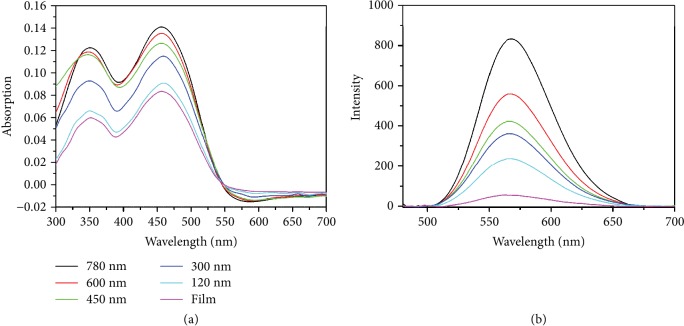
(a) Absorption and (b) fluorescence spectra collected on a flat film of DCM mixed with PMMA, and the nanohole arrays generated on the mixed film.

We tested this method with another dye molecule with red emission, 4-(dicyanomethylene)-2-methyl-6-(4-dimethylaminostyryl)-4H-pyran (DCM). The excitation wavelength of DCM is 462 nm, and the emission wavelength is 575 nm. The absorption spectra and fluorescence spectra of DCM also increase with increasing the depth of the nanohole arrays, as shown in Figure 6. The fluorescence intensity of DCM on a flat film is about 50 at the wavelength of 575 nm, which increases from 238 to 900 when the depth of nanoholes increases from 120 nm to 750 nm. The emission is enhanced for 18 times by using the 750 nm nanohole arrays.

**Figure 7:**
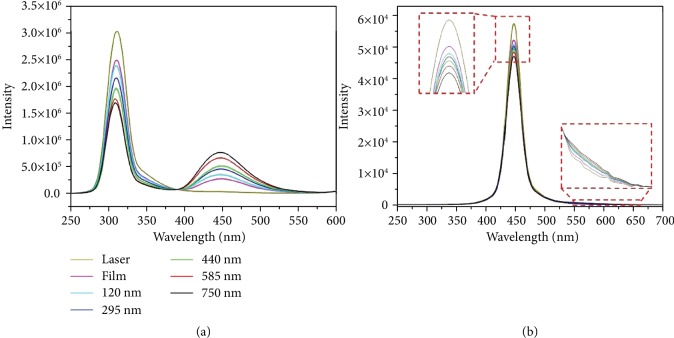
Excitation intensity and photoluminescence spectra collected on the nanohole arrays created on films of (a) TDPVBi and (b) DCM mixed with PMMA, a flat mixed film on a quartz slide is used as a reference.

Figure 7 shows the excitation intensity and photoluminescence spectra collected on a flat film of dye molecules mixed with PMMA and the nanohole arrays created on the film; the flat film with dye molecules on a quartz substrate was used as the reference. The measured fluorescence quantum efficiency is ~0.9 and 0.09 for TDPVBi and DCM, respectively, which is consistent (see Supporting Information, [Supplementary-material supplementary-material-1]). This result demonstrates that the antireflective arrays cannot enhance the fluorescence quantum efficiency of the fluorescent molecules. Therefore, the emission of fluorescent molecules is enhanced by increasing the absorption at the excitation wavelengths of the fluorescent molecules and the reduction of the fluorescent energy dissipation with antireflective arrays.

## 3. Conclusion

In summary, we demonstrate a universal and efficient method to enhance the emission of dye molecules by increasing the absorption and reducing the fluorescent energy dissipation using the antireflective arrays. The emission of TDPVBi and DCM is, respectively, enhanced for 14 and 18 times with the antireflective arrays. The patterned film of dye molecules mixed with PMMA not only works as an absorber but also works as an antireflective layer. This method is applicable for different molecules, which could be applied for enhancing the sensitivity of the sensing and imaging based on fluorescence.

## 4. Materials and Methods

### 4.1. Materials

All solvents and chemicals were of reagent quality and used without further purification. Ethanol, acetone, chloroform, and tetrahydrofuran (THF) were purchased from commercial sources in the highest available purity. Ultrapure water (18.2 M*Ω*·cm^−1^) was used directly from a Millipore System (Marlborough, France). The monodisperse polystyrene (PS) spheres with less than 5% diameter variation were obtained from Sigma-Aldrich. The Si wafers (n-type (100)) were obtained from Youyan Guigu Beijing, China. The poly-(methyl methacrylate) (PMMA), (heptadecafluoro-1,1,2,2-tetradecyl)trimethoxysilane and red fluorescent molecule 4-(dicyanomethylene)-2-methyl-6-(4-dimethylaminostyryl)-4H-pyran (DCM) were obtained from Sigma-Aldrich. The blue fluorescent molecule 2,5,20,50-tetrakis (2,2-diphenylvinyl)biphenyl (TDPVBi) was obtained from Alfa Aesar.

### 4.2. Fabrication of Si Stamps

The Si slides were cleaned by taking ultrasonication in a bath of acetone, chloroform, ethanol, and water for 5 min, respectively. The slides were then treated with oxygen plasma etching on a Plasma System 100 PVA Tepla, Germany, with O_2_ (100 mL/min) at a power density of 300 W for 3 min to make the substrates hydrophilic. Firstly, a monolayer of the hexagonal closed-packed PS spheres was prepared on Si (100) substrate (shown in [Supplementary-material supplementary-material-1]) according to the reference [30], which was utilized as a shadow mask for the subsequent RIE process. Secondly, the Si substrate with the PS spheres was subjected to the RIE process with SF_6_, CHF_3_, and O_2_ at a RF power of 100 W. The etching selectivity ratio of PS to Si is about 1 : 1.5. The Si nanopillar arrays of different heights were fabricated by adjusting the etching time from 3 min to 11 min. The processing gases were SF_6_ (30 sccm), CHF_3_ (6 sccm), and O_2_ (5 sccm), the radio frequency (RF) power was 100 W, and the chamber pressure was 30 mTorr. After lifting off the nanosphere mask, a tapered Si nanopillar array was created, whose periodicity depends on the size of the PS spheres.

### 4.3. Fabrication of Biomimetic Antireflective Arrays on Polymer Mixed with Dye Molecules

In a typical process, PMMA (*M*_W_ ≈ 96 k, 20 g) and dye molecules (DCM 0.1 g, TDPVBi 0.1 g) were dissolved in 200 mL of xylene by taking ultrasonication for 1 h. The quartz slides were cleaned by taking ultrasonication in a bath of acetone, chloroform, ethanol, and water for 5 min in turn. An 800 nm layer of PMMA mixed with dye molecules was spin-coated onto the quartz substrate, followed by baking at 120°C for 5 min. Subsequently, the fabricated silicon stamp was imprinted on the spin-coated layer of PMMA mixed with dye molecules with a pressure of 40 bars at 170°C. After peeling off the stamp from the substrate at 70°C, the pattern of the stamp was transferred onto the substrate. Finally, the biomimetic antireflective array was created with a film of PMMA mixed with dye molecules on a quartz substrate.

### 4.4. Characterization

SEM micrographs were taken with JEOLJSM6700F field emission SEM with a primary electron voltage of 3 kV; the samples were sputtered with a layer of Pt (2 nm thickness) before imaging to improve the conductivity. Spectra were collected on a spectroscopy meter (Shimadzu UV3600, Shimadzu, Japan). The UV-VIS absorption spectra of films were carried out using the hemispherical measurements with an integrating sphere (Ocean sphere). A xenon lamp (PLS-SXE300) was coupled to a fiber optic spectrometer (Maya 2000), which was used for wavelength-dependent measurement at a waveband of 300-1200 nm. The sample was mounted in the center of the integrating sphere. The reflected and transmitted light from the sample was uniformly scattered by the integrating sphere and collected by a photodetector. The schematic illustration of the spherical measurement is shown in [Supplementary-material supplementary-material-1]. In the measurements, we accounted for all light reflected from and transmitted through the sample, so this can be considered as a measurement of the absolute absorption. The reflectivity measurement was conducted on a spectrometer attached to standard mirror reflection optics at the incidence angle of 5 degrees. Fluorescence measurements were carried out with RF-5301PC instrument. The absolute fluorescence quantum yield was measured by using a Hamamatsu quantum yield spectrometer C11347 Quantaurus-QY.

## Supplementary Materials

Supplementary Materials Fig. S1: SEM image of the monolayer of nanospheres. The scale bar represents1 *μ*m. Fig. S2: cross-sectional SEM images of the Si stamps with a height of (A) 120, (B) 300, (C) 450, (D) 600, and (E) 780 nm; (F) hemispherical reflection of the above Si stamps and a flat Si slide at normal incidence. Fig. S3: the setup for measuring the spectra with an integrating sphere. Table S1: fluorescence quantum efficiency of different dye molecules on the flat films and nanohole arrays of different depths.
