# Supplementary material for: Emission Enhancement of Fluorescent Molecules by Antireflective Arrays
Source: Research (Wash D C). 2019 Nov 27;2019:3495841. doi: 10.34133/2019/3495841 (PMC6944513; doi:10.34133/2019/3495841)
Supplement: Supplementary Materials — Fig. S1: SEM image of the monolayer of nanospheres. The scale bar represents1 μm. Fig. S2: cross-sectional SEM images of the Si stamps with a height of (A) 120, (B) 300, (C) 450, (D) 600, and (E) 780 nm; (F) hemispherical reflection of the above Si stamps and a flat Si slide at normal incidence. Fig. S3: the setup for measuring the spectra with an integrating sphere. Table S1: fluorescence quantum efficiency of different dye molecules on the flat films and nanohole arrays of different depths. [file 3495841.f1.docx]

Supplementary Materials

Emission Enhancement of Fluorescent Molecules by Antireflective Arrays

Hongbo Xu^1,2^, Lingxiao Liu^2^, Fei Teng^2^, Nan Lu^2*^

1: MIIT Key Laboratory of Critical Materials Technology for New Energy Conversion and Storage, School of Chemistry and Chemical Engineering, Harbin Institute of Technology,150001, Harbin, China.

2: State Key Laboratory of Supramolecular Structure and Materials, College Chemistry, Jilin University130012, Changchun, P. R. China

Emails: luenan@jlu.edu.cn


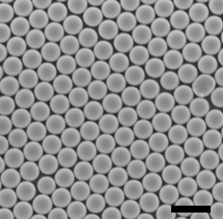


**Fig. S1** SEM image of the monolayer of nanospheres. The scale bar represents1 µm.


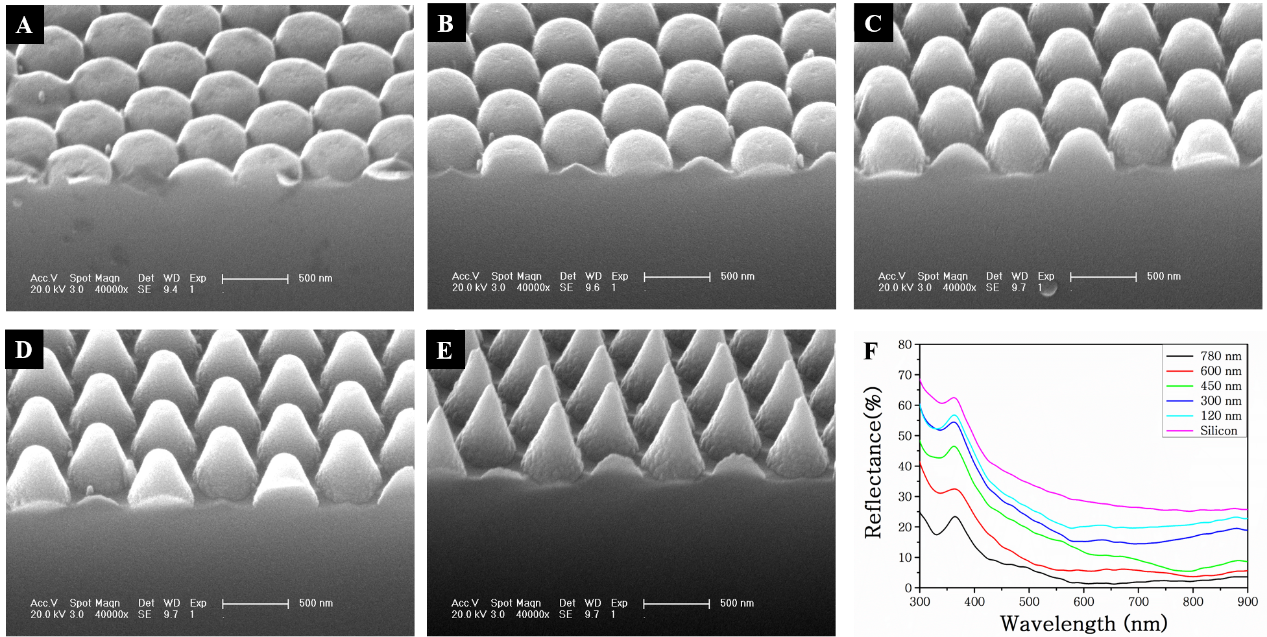


**Fig. S2** Cross-sectional SEM images of the Si stamps with height of (A) 120, (B) 300, (C) 450, (D) 600 and (E) 780 nm; (F) Hemispherical reflection of the above Si stamps and a flat Si slide at normal incidence.


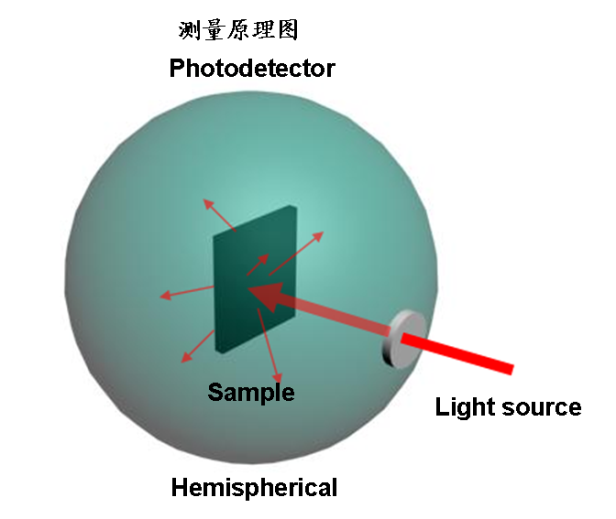


**Fig. S3** The setup for measuring the spectra with an integrating sphere.

**Table S1** Fluorescence quantum efficiency of different dye molecules on the flat films and nanohole arrays of different depths.

| Sample | film | 120 nm | 295 nm | 440 nm | 585 nm | 750 nm |
| --- | --- | --- | --- | --- | --- | --- |
| TDPVBi | 0.89 | 0.92 | 0.90 | 0.89 | 0.88 | 0.91 |
| DCM | 0.097 | 0.095 | 0.098 | 0.094 | 0.091 | 0.094 |
